# Immunogenic senescence sensitizes lung cancer to LUNX-targeting therapy

**DOI:** 10.1007/s00262-021-03077-1

**Published:** 2021-10-21

**Authors:** Defeng Jiao, Xiaohu Zheng, Xianghui Du, Dong Wang, Ziming Hu, Rui Sun, Zhigang Tian, Binqing Fu, Haiming Wei

**Affiliations:** 1 Division of Molecular Medicine, Hefei National Laboratory for Physical Sciences at Microscale, The CAS Key Laboratory of Innate Immunity and Chronic Disease, School of Life Sciences, University of Science and Technology of China, Hefei, 230027 China; 2 Institute of Immunology, University of Science and Technology of China, Hefei, 230027 China

**Keywords:** Immunogenic chemotherapy, Senescence, LUNX, Antibody, Lung cancer

## Abstract

The higher immunogenicity of tumors usually predicts favorable therapeutic responses. Tumor antigens dominate the immunogenic character within tumors. We investigated if there was a targetable tumor antigen during immunogenic chemotherapy within lung cancer. Chemotherapy-induced immunogenic senescence was demonstrated using a multi-marker, three-step workflow, and RNA-sequencing data. The ability of anti-lung-specific X protein (LUNX) antibody to suppress the survival of senescent lung cancer cells was evaluated in vitro and in vivo using real-time cytotoxicity analysis and xenograft mouse models, respectively. The induction of cellular senescence by immunogenic chemotherapy boosted cell-surface shuttling of LUNX and enhanced the immunogenic features of senescent tumor cells, which sensitized lung cancer cells to anti-LUNX antibody-mediated therapy and contributed to tumor suppression. The immunogenic senescence-mediated anti-tumor response was triggered by the direct action of antibody on tumor cells, strengthened by natural-killer cells through an antibody-dependent cell-mediated cytotoxicity response, and ultimately, led to tumor control. Our findings suggest that LUNX is a lung cancer targetable-immunogenic antigen. The proportion of lung cancers responding to LUNX-targeting therapy could be expanded substantially by immunogenic chemotherapy that induces senescence-associated translocation of LUNX to the plasma membrane.

**Supplementary Information:**

The online version contains supplementary material available at 10.1007/s00262-021-03077-1.

## Introduction

Recent studies investigating tumor immunogenicity have, in general, focused on cell-intrinsic damage-associated molecular patterns, such as the exposure of calreticulin on the cell surface, and the release of adenosine triphosphate and high-mobility group box 1 into the extracellular environment [17, 25]. The aberrant location of these molecules exhibits strong adjuvanticity and alerts the immune system to “a dangerous state” being present [30, 38]. However, these molecules alone are not sufficient to initiate an adaptive immune response, and they are usually normal cellular components carrying out crucial metabolism or structural functions [16, 33]. Hence, none of these molecules is a truly specific immunogenic tumor antigen. As immunogenicity of tumors depends on both adjuvanticity and antigenicity, defining tumor-specific immunogenic antigens during immunogenic chemotherapy can be a very interesting and rational task.

The lung-specific X protein (LUNX) gene (also known as BPIFA1, SPLUNC1, and PLUNC) was isolated first by Kyoko and colleagues [22]. They established a LUNX-based real-time reverse transcription-quantitative polymerase chain reaction (RT-qPCR) assay for detection of micro-metastases in the lymph nodes of non-small-cell lung cancer (NSCLC) patients. It has also been reported that LUNX protein levels in normal small airways are relatively low, and LUNX staining is absent in normal peripheral lung tissue [3, 5]. A moderate increase in LUNX expression in nasal secretions and sputum has been detected to occur in inflammatory airway diseases such as allergic rhinitis and chronic obstructive pulmonary disease [13, 27]. Subsequent reports have suggested that LUNX is undetectable in the normal adrenal gland, brain, liver, kidney, and peripheral lung tissues in humans [4, 5, 22]. Building on such work, our previous study proposed LUNX mRNA and LUNX protein to be a specific diagnostic marker and a novel candidate therapeutic target for NSCLC, respectively, because no detectable expression of LUNX has been found in other tumors such as hepatocellular carcinoma or ovarian cancer [10, 42]. LUNX overexpression promotes the migration and proliferation of lung cancer cells and, in general, predicts a poor prognosis for NSCLC patients. Targeting LUNX via anti-LUNX antibody or LUNX-chimeric antigen receptor (CAR) T cells has been shown to suppress the growth and metastasis of xenografts and patient-derived xenografts in a mouse model of NSCLC [20, 42]. Mechanismly, upon binding to the surface LUNX, the anti-LUNX antibody reduces LUNX protein levels via antibody-mediated endocytosis and degradation which in turn represses downstream signaling responsible for tumor cell proliferation and survival [42]. Therefore, LUNX might represent a promising targetable antigen for NSCLC.

“Cellular senescence” is a stable growth-arresting cellular state triggered by nonlethal stress, such as exposure to oxidative damage or genotoxic agents, overexpression of oncogenes, or mitochondrial dysfunction [2, 18]. Alessandra and colleagues showed that myeloma cells treated with therapeutic agents such as melphalan up-regulated the expression of DNAX accessory molecule-1 and natural killer group 2D (NKG2D) ligands and exhibited a senescent-like phenotype [39]. Other reports have also suggested that senescent cells could secrete a wide range of senescence-associated secretory phenotypes (SASPs), which could modulate the immune response and have direct effects on tumor progression [12, 15].

Here, we report that immunogenic chemotherapy-treated lung cancer cells exhibited an immunogenic senescence phenotype and had high expression of LUNX on their plasma membrane, which served as a targetable immunogenic antigen. Targeting LUNX on cells membranes via anti-LUNX antibody could suppress the growth of lung cancer cells significantly *in vitro* and *in vivo*. Our study suggests that: (1) LUNX is a lung cancer-targetable tumor antigen during immunogenic senescence; (2) combining anti-LUNX antibody with immunogenic drugs that can induce senescence-associated translocation of LUNX to the plasma membrane might offer a new strategy to treat NSCLC.

## Materials and methods

### Mice

Experimental procedures involving animals were conducted in accordance with the National Guidelines for Animal Usage in Research (China). Permission to undertake animal studies was obtained from the Ethics Committee of the University of Science & Technology of China (Hefei, China).

Female BALB/cJGpt-Foxn1^nu^/Gpt and NOD/ShiLtJGpt-Prkdc^em26Cd52^Il2rg^em26Cd22^/Gpt mice (aged 6 weeks) were purchased from GemPharmatech (Nanjing, China). Mice were maintained under specific pathogen-free conditions.

### Cell lines

A549 cells and NCI-H292 cell lines were obtained from the Shanghai Cell Bank (Chinese Academy of Sciences, Shanghai, China). They were passaged by our research team for < 6 months after receipt or revival. Cells were cultured in complete RPMI medium 1640 (HyClone, Logan, UT, USA) with 10% fetal bovine serum (Gibco, Grand Island, NY, USA) plus 1% streptomycin and penicillin in an atmosphere of 5%CO_2_ at 37 °C. The average diameter of A549 cells and NCI-H292 cells was measured using the Countstar® cell-analysis system (ALIT Life Sciences, Shanghai, China) after cells had been trypsinized.

### Antibody production

The anti-LUNX antibody was introduced in our previous work [42] and humanized to replace the Fc region with the human IgG1 Fc region. Briefly, S-35-8 (IgG2a-k) LUNX hybridoma was raised against a His-tagged LUNX protein and screened by ELISA. The hybridoma was cultured in RPMI-1640 medium. The S-35-8 anti-LUNX antibody was purified by protein A affinity chromatography (GE Healthcare Bio-Sciences AB).

### Immunofluorescence

A549 cells and NCI-H292 cells were plated at 1 × 10^5^ onto 15-mm glass-bottom culture dishes overnight. They were treated with the indicated concentration of mitoxantrone or vehicle for 48 h. After removal of culture supernatants, cells were washed twice with cold phosphate-buffered saline (PBS) and blocked for 30 min at room temperature (RT) with 1% bovine serum albumin plus 5% normal goat serum. Primary antibody and secondary antibody were added for 60 min at 4 °C, followed by fixation with 4% paraformaldehyde for 15 min and staining with 4',6-Diamidino-2-phenylindole dihydrochloride for 7 min at RT. Next, cells were washed thrice with cold PBS and visualized using a confocal laser scanning microscope (LSM 880; Zeiss, Oberkochen, Germany). For intracellular staining of HP1γ, cells were permeabilized with 0.1% Triton-X for 10 min at RT after staining cell surface expression of LUNX.

### Flow cytometry

A549 cells and NCI-H292 cells were trypsinized and collected by centrifugation (125 × *g*, 5 min, RT). After resuspension in 100 μL of PBS containing the defined antibody, cells were incubated for 30 min at 4 °C. Then, the cells were washed twice with cold PBS and investigated by flow cytometry (LSRII; BD Biosciences, Franklin Lakes, NJ, USA).

### RT-qPCR

Total RNA was prepared using TRIzol® Reagent (Invitrogen, Carlsbad, CA, USA) and reverse-transcribed into complementary (c) DNA using a random primer (Invitrogen). The resulting cDNA was analyzed for the expression of LUNX, UL16-binding protein (ULBP) 1–6, MIC-A, and MIC-B. Real-time PCR was carried out in triplicate using SYBR Green, and *β*-actin served as an endogenous normalization control.

### Senescence-associated *β*-galactosidase staining

A549 cells and NCI-H292 cells were plated at 1 × 10^5^ in six-well culture plates overnight. They were treated with the indicated concentration of mitoxantrone or vehicle for 48 h. After removal of culture supernatants, cells were washed twice with cold PBS and fixed for 15 min at RT. Then, cells were incubated with fresh senescence-associated *β*-galactosidase (SA-*β*-gal) staining solution (Beyotime Institute of Technology, Beijing, China) overnight without CO_2_ at 37 °C. If needed, cells were trypsinized moderately to show the edge of each cell before fixation. Images were acquired using a microscope (IX-81; Olympus, Tokyo, Japan).

### Western blotting

Lysates of whole A549 cells and NCI-H292 cells were prepared using RIPA Lysis and Extraction Buffer (Thermo Scientific, Waltham, MA, USA) supplemented with Halt Protease Inhibitor Cocktail (Thermo Scientific). After centrifugation (14,000 × *g*, 10 min, 4 °C), supernatants were collected for sodium dodecyl sulfate–polyacrylamide gel electrophoresis. Then, proteins were transferred to polyvinylidene difluoride (PVDF) membranes and blocked with QuickBlock™ Western for 15 min at RT. PVDF membranes were incubated with primary antibodies overnight at 4 °C and then incubated with horseradish peroxidase-conjugated secondary antibodies for 1 h at RT. Protein bands were developed by chemiluminescence autoradiography.

### Enzyme-linked immunosorbent assay (ELISA)

Measurement of the level of interleukin (IL)-6 and IL-8 in culture supernatants was carried out using ELISAs according to manufacturer instructions. Briefly, culture supernatants were added to pre-coated 96-well plates and incubated with detection antibody for 60 min at RT. Then, the plates were washed thrice and incubated with streptavidin–horseradish peroxidase for 20 min at RT. 3,3′,5,5′-Tetramethylbenzidine substrate solution was added. The reaction was stopped after 2–5 min followed by measurement of absorbance in 96-well plates at 450 nm.

### Real-time cytotoxicity assays

The cytotoxicity assays of A549 cells and NCI-H292 cells were monitored using the xCELLigence Real-Time Cell Analyzer-Multiple Plate system (Roche Applied Science, Basel, Switzerland). Briefly, normal cells or senescent A549 cells and NCI-H292 cells were plated at 1 × 10^4^ in 16-well *E*-plates for ~ 20 h until the Cell Index reached 1. Then, freshly isolated natural killer (NK) cells from human peripheral blood mononuclear cells (PBMCs) or anti-LUNX antibody were added to the *E* plate at a defined effect/target ratio or concentration, respectively. For real-time monitoring, the Cell Index was read automatically every 15 min.

### Isolation of NK cells from human PBMCs

Human PBNCs were isolated using Ficoll density gradients. NK cells were purified by a Magnetic-Activated Cell Sorter kit (Miltenyi Biotec, Bergisch Gladbach, Germany). The purity of NK cells for each assay was > 90%.

### *In vivo* experiments

Female nude or NCG mice were used to establish subcutaneous and metastatic lung cancer xenografts to assess the antitumor capacity of a combination of chemotherapy and anti-LUNX antibody. For the subcutaneous xenograft model, 2 × 10^6^ A549 cells were injected (s.c.) into the axilla and treatment started once the tumors were palpable. The tumor volume was calculated as (width^2^ × length) / 2. In the metastatic xenograft model, 5 × 10^5^ A549-luciferase cells were transplanted (i.v.) into mice. After 24 h, mice were assigned randomly to groups and treated as indicated. Tumor metastasis was analyzed by live body images of mice.

### Statistical analyses

Statistical significance was determined using Prism 8.0 (GraphPad, San Diego, CA, USA). Two-tailed unpaired Student’s *t*-tests between two groups and one-way analysis of variance across multiple groups were used to determine significance. A one-sided Fisher’s exact test was utilized to estimate the correlation between the expression of LUNX and Heterochromatin protein 1 homolog gamma (HP1*γ*). Data represent the mean ± SD. *P* < 0.05 was considered significant.

## Results

### Immunogenic chemotherapy induces translocation of LUNX to the plasma membrane

**Fig. 1:**
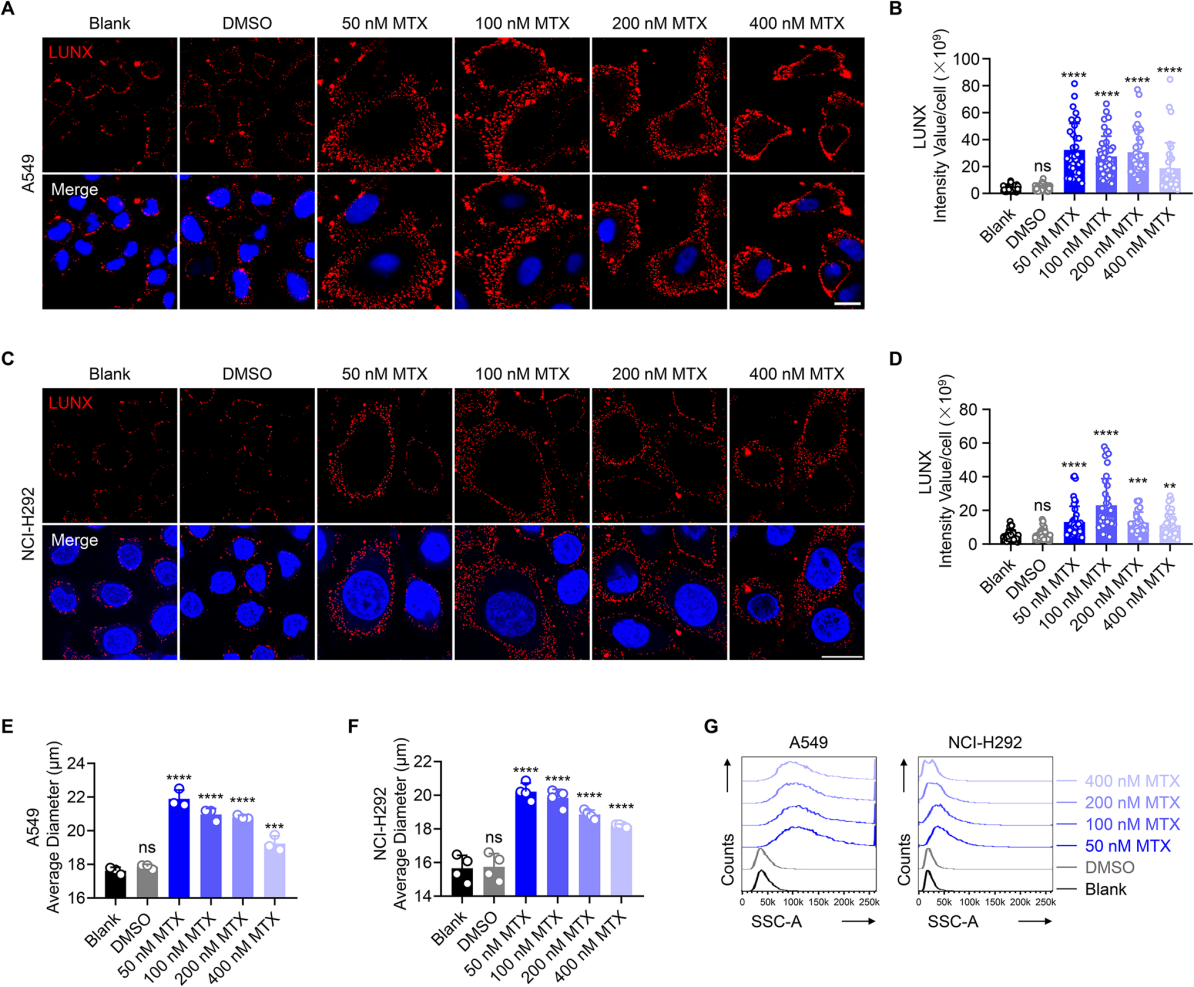
Cell-surface shuttling of LUNX after immunogenic chemotherapy (**A**–**D**) Surface exposure of LUNX was determined by immunofluorescence 48 h after treatment with the indicated concentration of mitoxantrone (MTX). Representative images are shown for A549 (**A**) and NCI-H292(**C**). Scale bar = 20 μm. We calculated the intensity value for each A549 cell (B) and NCI-H292 cell (**D**). Anti-LUNX concentration = 200 μg/mL. (**E**, **F**) The average diameter of A549 (**E**) and NCI-H292 (**F**) 48 h after treatment with mitoxantrone. (*n* = 3–4 replicates). (**G**) Flow cytometry for side-scatter detection of A549 cells and NCI-H292 cells 48 h after mitoxantrone treatment

Immunogenic tumors exhibit a better prognosis than non-immunogenic tumors [36]. Hence, we examined the ability of several Food and Drug Administration (FDA)-approved chemotherapeutic agents to induce cell-surface shuttling of LUNX, which we proposed previously to be a promising tumor antigen in NSCLC [32, 42]. The first-line chemotherapy agent cisplatin slightly stimulated the translocation of LUNX to the cell surface at the maximum dose tested (Figure S1A, B). Nevertheless, gemcitabine treatment stimulated cell-surface translocation of LUNX at all doses tested (Figure S1C, D). Furthermore, the anthracycline mitoxantrone, which can show an immunogenic effect [30], strongly increased the LUNX expression on the surface of A549 cells and NCI-H292 cells after 48 h of treatment, especially at 50 nM and 100 nM (Fig. 1A–D). To exclude the possibility that the increased expression of LUNX on the cell surface was due to transcriptional up-regulation of LUNX mRNA, we evaluated LUNX expression at the mRNA level (Figure S1E–G). There was no significant difference in mRNA expression between all doses tested for mitoxantrone, cisplatin, or gemcitabine. Total protein level expression of LUNX also exhibited no significant change after mitoxantrone treatment (Figure S1H). Intriguingly, we observed a significant increase in the cell volume upon immunofluorescence staining (Fig. 1A, C) and measured by average diameter of both cell types (Fig. 1E, F). Flow cytometry revealed not only the enlarged cell volume (data not shown) but increased cytoplasmic granularity as demonstrated by a higher side scatter-A strength (Fig. 1G). Taken together, these data suggested that immunogenic chemotherapy strongly stimulated the translocation of LUNX to the plasma membrane and that this phenomenon might be related to a significant change in cell morphology.

### Cellular senescence correlates with LUNX shuttling

**Fig. 2:**
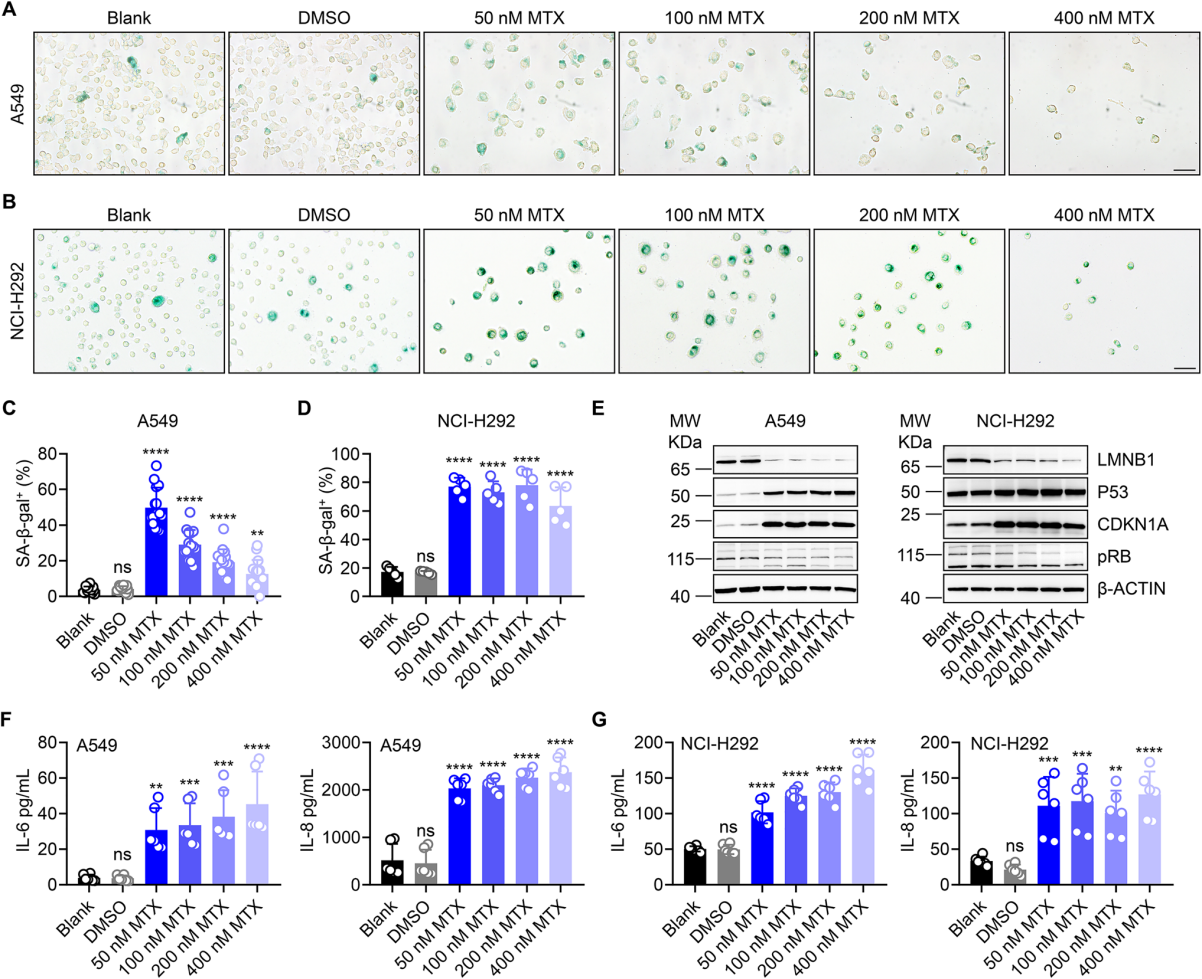
Induction of cellular senescence **A**–**D** A549 cells and NCI-H292 cells were assayed for SA-b-gal activity 48 h after treatment with the appropriate concentration of mitoxantrone. Representative images are shown for A549 cells (**A**) and NCI-H292 cells (**C**). Scale bar = 50 μm. We calculated the ratio of SA-*β*-gal-positive A549 cells (**B**) and SA-*β*-gal-positive NCI-H292 cells (**D**). **E** Lysates of whole A549 cells and NCI-H292 cells were collected 48 h after treatment with mitoxantrone and analyzed by western blotting using the indicated antibodies. Actin is shown as a loading control. (**F**, **G**) *In vitro* release of IL-6 and IL-8 by A549 cells (**F**) and NCI-H292 cells (**G**) in response to mitoxantrone as determined by ELISA

We wished to determine the mechanism by which mitoxantrone treatment promoted LUNX translocation and induced a flattened, enlarged morphology. Hence, we focused on investigating the significant change in cell morphology after mitoxantrone treatment [14]. As reported previously, mitoxantrone treatment leads to the immunogenic cell death of tumor cells, which is a *bona fide* apoptotic form of cell death, but without the morphological change mentioned above [12, 40]. Seifrtova and colleagues reported that exposure of dental pulp stem cells and human dermal fibroblasts to mitoxantrone resulted in cellular senescence that was characterized by the typical morphological changes we mentioned above [37]. Hence, we first analyzed the expression of SA-*β*-gal in tumor cells after mitoxantrone treatment, which serves as the most classical marker of senescence [18]. A549 cells and NCI-H292 cells showed a sharp increase in SA-*β*-gal activity, with positive cells exhibiting a flattened, enlarged morphology (Fig. 2A–D). Moreover, these cells maintained and increased the expression of SA-*β*-gal 7 days after drug withdrawal (Figure S2A, B). Analyses of protein expression confirmed the dramatic reduction of LMNB1 expression (Fig. 2E), which is another marker of senescence [18]. The P53-P21 (also known as cyclin-dependent kinase inhibitor 1) pathway and retinoblastoma-associated protein pathway are known to be “master regulators” of the senescence program [11]. Mitoxantrone treatment significantly stimulated the expression of P53 and its downstream target P21. The protein expression of pRB decreased slightly (Fig. 2E). SASP components, including proinflammatory and immune-modulatory cytokines such as IL-6 and IL-8, are also key features of senescence [8]. By measuring the expression of IL-6 protein and IL-8 protein in the cell-culture supernatants of mitoxantrone-treated A549 cells and NCI-H292 cells, we discovered a significant 2–6 fold increase in both cell lines tested when compared with that in a non-treated control (Fig. 2F, G). Besides, western blotting detecting the cleaved caspase 3 in A549 and NCI-H292 cells treated with low dose of mitoxantrone also showed no obvious apoptosis (Fig S2C and D). FACS data also revealed that only a few portion of cells died after low dose of mitoxantrone treatment in both A549 and NCI-H292 cells as showed by annexin V and 7AAD staining (Fig S2E and F). Our data indicated that the low dose of mitoxantrone treatment did not induce apoptosis in A549 and NCI-H292 cells. These results suggested that mitoxantrone treatment induced A549 cells and NCI-H292 cells to undergo senescence.

**Fig. 3:**
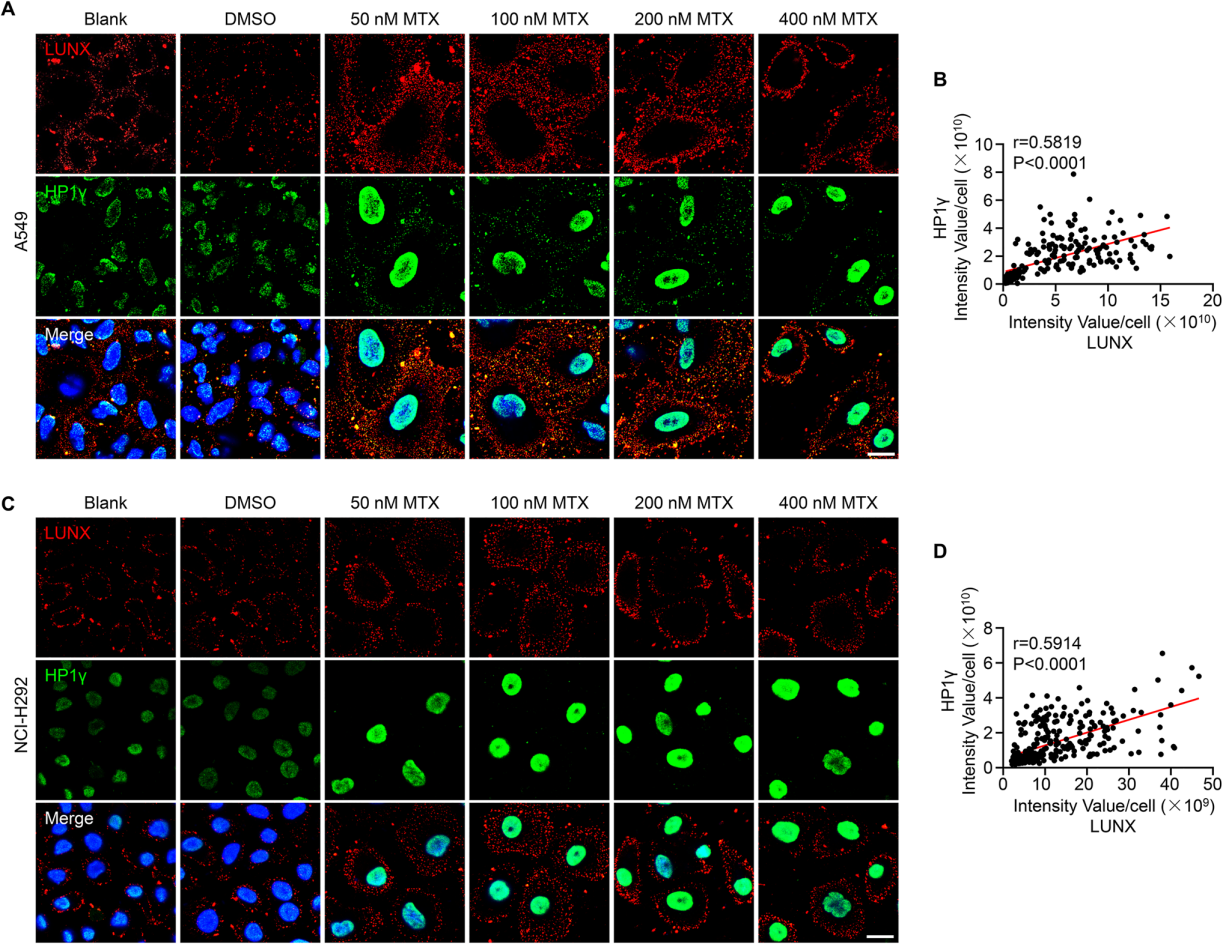
Cellular senescence correlates with LUNX shuttling (**A**–**D**) Confocal laser scanning microscopy showing the expression of LUNX (red) and HP1γ (green) in A549 cells (**A**) and NCI-H292 cells (**C**). Scale bar = 20 μm. Anti-LUNX concentration = 200 μg/mL. Correlation between exposure to LUNX of the cell surface and nuclear expression of HP1γ in A549 cells (**B**) and NCI-H292 cells (**D**)

We wished to test directly whether cell-surface shuttling of LUNX was associated with cellular senescence induced by mitoxantrone. Hence, we co-stained LUNX with HP1γ, expression of which has also been shown to increase during senescence [29]. Mitoxantrone treatment led to a significant increase in the expression of LUNX and HP1*γ* (Fig. 3A, B). Furthermore, there was a strong correlation between the surface exposure of LUNX and the nuclear expression of HP1*γ* (Fig. 3B, D). These data provided evidence that mitoxantrone treatment-induced A549 cells and NCI-H292 cells to undergo senescence, which promoted the translocation of LUNX to the plasma membrane.

### Senescent cells exhibit an “immunogenic signature”

**Fig. 4:**
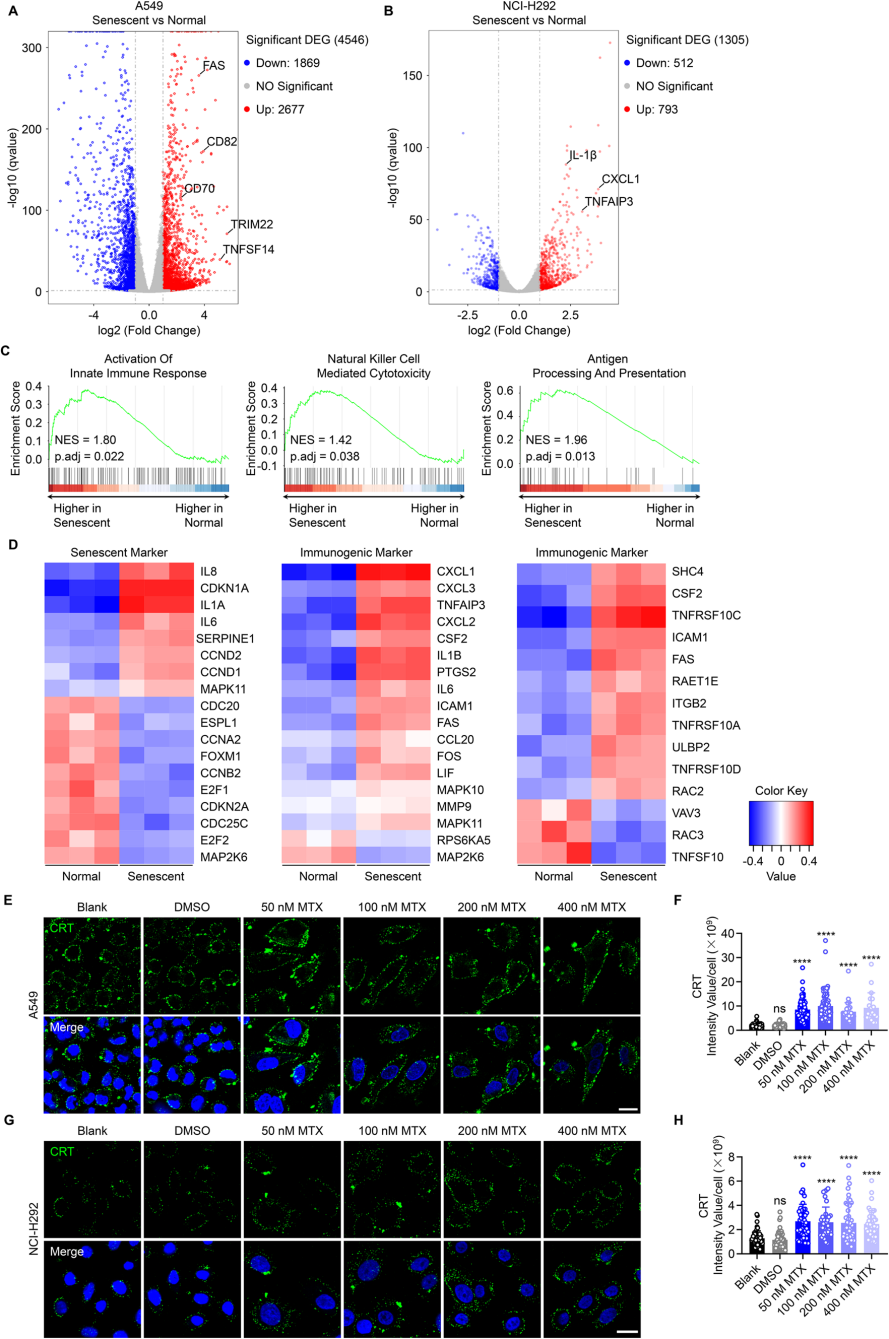
Senescent cells exhibit an immunogenic signature (**A**, **B**) Volcano plots of RNA-seq transcriptional profiles after treatment with 50 nM mitoxantrone for 48 h in A549 cells (**A**) and NCI-H292 cells (**B**). Genes that show significantly different expressions are shown. **C** Significantly enriched immunogenic pathways according to gene set enrichment analysis comparing mitoxantrone-treated A549 cells *versus* normal A549 cells. **D** Expression of signature core cellular senescence genes and immunogenic genes in mitoxantrone-treated NCI-H292 cells *versus* normal NCI-H292 cells. **E**–**H** Confocal laser scanning microscopy of the expression of calreticulin on the surface of A549 cells (**E**) and NCI-H292 cell (**G**) treated with the indicated concentration of mitoxantrone. We calculated the intensity value of each A549 cells (**F**) and NCI-H292 cell (**H**). Scale bar = 20 μm

We identified that LUNX translocation correlated strongly with the senescence of tumor cells induced by mitoxantrone treatment. Next, we aimed to evaluate the exact differences between normal cells and senescent tumor cells by RNA sequencing of normal (dimethyl sulfoxide-treated) and senescent (mitoxantrone-treated) A549 cells and NCI-H292 cells. Collectively, up-regulation of expression of 2677 unique transcripts and down-regulation of 1869 transcripts was evident in the transcriptional profiles of senescent A549 cells compared to normal A549 cells (Fig. 4A). Nevertheless, there were far fewer and less significant transcripts in senescent NCI-H292 cells compared with those in normal NCI-H292 cells, including only 793 up-regulated transcripts and 512 down-regulated transcripts (Fig. 4B). The expression of transcripts encoding the cell-surface markers of activation (e.g., Tumor necrosis factor ligand superfamily member 14 (TNFSF14) and cluster of differentiation-70) and inflammatory cytokines (e.g., IL-1*β* and CXCL1) was also up-regulated (Fig. 4A, B) [19, 23]. The elevated expression of these genes indicated that senescent tumor cells might recruit and activate immune cells more potentially.

Analyses of mRNA transcripts using the Kyoto Encyclopedia of Genes and Genomes revealed that genes were categorized in different signal-transduction pathways between A549 cells and NCI-H292 cells (Figure S3A, B), but the “Cellular senescence” pathway, “Cellular cycle” pathway, and “P53 signaling” pathway were enriched in both. We undertook gene set enrichment analysis of canonical pathways curated by the MSig database. Comparison of the transcriptomes of senescent *versus* normal A549 cells (Figure S3C) or senescent *versus* normal NCI-H292 cells (Figure S3D) showed that the G2M checkpoint, E2F targets, and Myc targeting pathways were enriched in normal cells but not in senescent cells. Hence, cell-cycle arrest and proliferation of stasis in senescent A549 cells and NCI-H292 cells occurred, which confirmed the effect of mitoxantrone-induced senescence [9].

Intriguingly, in senescent A549 cells, the “Activation of the Innate Immune Response’’ pathway was enriched significantly, as was the “Natural Killer Cell-Mediated Cytotoxicity’’ pathway, which suggested that senescent tumor cells might activate an NK cell-mediated response (Fig. 4C). Senescent NCI-H292 cells exhibited similar signature (Figures S3D). Further evidence for the translocation of LUNX was the observation that the “Antigen Processing And Presentation’’ pathway and “Protein Secretion’’ pathway were also enriched in A549 cells and NCI-H292 cells, respectively (Fig. 4C, S3D). Heatmap analyses revealed expression of pivotal senescent genes (left column) and immunogenic genes enriched in TNF pathway (middle column) and natural killer cell-mediated cytotoxicity pathway (right column) such as IL-6, IL-8, CDKN1A, ICAM1, FAS, and ULBP2 (Fig. 4D). Unexpectedly, the expression of a previously reported canonical marker of immunogenic cell death, calreticulin, was also enriched in the cellular senescence pathway of the Gene Ontology database (data not shown) [33]. To confirm this observation, we measured the calreticulin expression on A549 cells and NCI-H292 cells after mitoxantrone treatment by immunofluorescence. Mitoxantrone treatment increased the level of cell-surface calreticulin markedly, which might help the translocation of LUNX and the increasing immunogenicity (Fig. 4E–H). These findings indicated that immunogenic chemotherapy treatment-induced cellular senescence and that these senescent tumor cells exhibited an immunogenic signature.

### Immunogenic senescent cells are sensitive to LUNX-targeting therapy

**Fig. 5:**
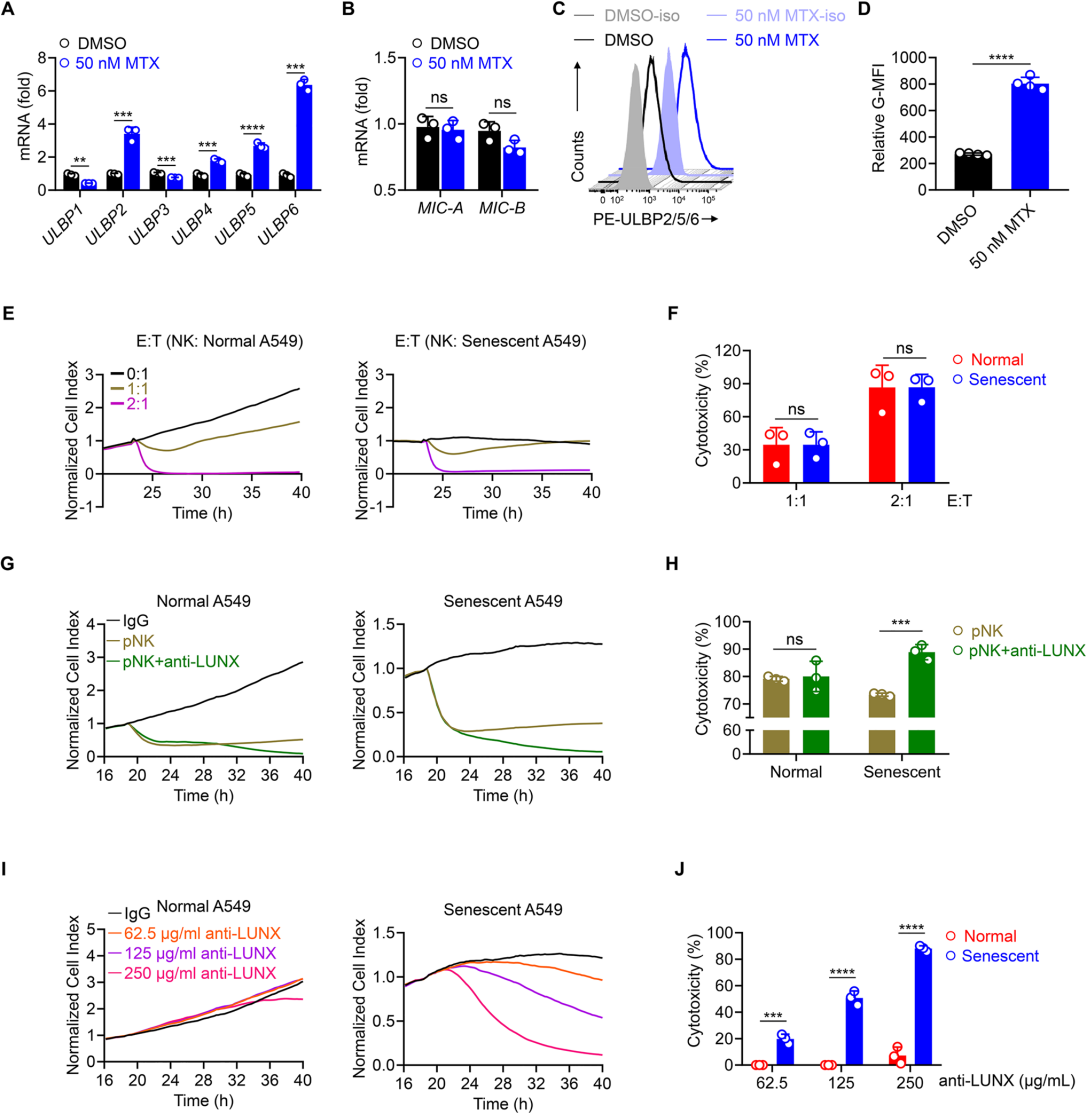
Immunogenic senescent cells are more sensitive to LUNX-targeting therapy (**A**, **B**) RT-qPCR of the genes of NKG2D ligands in A549 cells treated with mitoxantrone or DMSO. **C**, **D** Flow cytometry showing ULBP2/5/6 expression in A549 cells treated with mitoxantrone or DMSO. We calculated the relative geometric mean fluorescence intensity of A549 cells treated with mitoxantrone or DMSO. **E**, **F** Real-time Cell Index measurement of normal or senescent (mitoxantrone-treated) A549 cells as target cells cultured with different ratios of effector NK cells. The relative cytotoxicity is shown in (**F**). **G**, **H** Real-time Cell Index measurement of normal or senescent A549 target cells cultured at a ratio 2:1 of effector NK cells with or without 250 μg/mL anti-LUNX antibody. The relative cytotoxicity is shown in (**H**). **I**, **J** Real-time Cell Index measurement of normal or senescent target A549 cells cultured in the presence of different concentrations of anti-LUNX antibody alone. The relative cytotoxicity is shown in (J)

We employed RT-qPCR to ascertain if expression of known NKG2D ligands was up-regulated, as shown by transcription data (Fig. 4D). Human NKG2D ligands consist of the ULBP family, including ULBP1-6, MIC-A, and MIC-B [6] [35]. Of note, a more-than-twofold increase in the expression of ULBP2, ULBP5, and ULBP6 was observed, whereas the expression of ULBP1 and ULBP3 decreased. There was no significant difference in the expression of MIC-A and MIC-B after mitoxantrone treatment (Fig. 5A, B). Furthermore, flow cytometry revealed that the relative geometric mean fluorescence intensity of ULBP2/5/6 was much higher in mitoxantrone-induced senescent A549 cells compared with that in normal A549 cells (Fig. 5C, D). Similar results were observed in NCI-H292 cells (Figure S4A–D).

Having identified the increased expression of NK-cell activating ligands in senescent A549 cells, we hypothesized that senescent A549 cells might be more sensitive to NK cell-mediated killing [34]. Using real-time cytotoxicity analysis (RTCA) of normal cells alone or senescent A549 cells alone, we found that normal A549 cells showed exponential growth whereas senescent A549 cells barely proliferated at the indicated time points, which reflected the basic difference between normal cells and senescent cells (Fig. 5E). Upon co-culture with NK cells at different effect/target ratios, normal cells or senescent A549 cells were killed rapidly but showed no significant difference in cytotoxicity at the same E/T ratio (Fig. 5E, F).

Early reports suggested that NK cells specifically target tumor cells undergoing therapy-induced senescence in an NKG2D ligand-dependent manner, but this effect might not be true for mitoxantrone-induced senescent A549 cells [7]. Another important signature of senescent A549 cells and NCI-H292 cells demonstrated previously was the substantial expression of LUNX protein on cell membranes, which could be targeted as lung cancer-specific antigen by anti-LUNX antibody (Fig. 1A–D). Accordingly, we tested whether anti-LUNX antibody could promote NK cell-mediated cytotoxicity towards senescent A549 cells. RTCA data revealed that senescent A549 cells co-cultured with NK cells in the presence of anti-LUNX antibody showed a stronger killing efficiency than those co-cultured without anti-LUNX antibody after 24 h. However, when using normal A549 cells, a significant difference in the normalized Cell Index was not found until 36 h (Fig. 5G, H). This result suggested that, compared with normal A549 cells, senescent A549 cells might respond more strongly to anti-LUNX antibody. Hence, we measured the ability of anti-LUNX antibody alone to suppress the survival of A549 cells.

Increasing concentrations of anti-LUNX antibody exhibited no or a moderately inhibitory effect upon normal A549 cells. However, senescent A549 cells showed much greater sensitivity to treatment with anti-LUNX antibody alone without NK cells, as shown by a significantly decreased normalized Cell Index, which was dependent upon the concentration (Fig. 5I, J). Although senescent NCI-H292 cells also up-regulated the expression of LUNX, increasing sensitivity to antibody treatment was not observed in these senescent cells. This result might have been because the unrestrained proliferation of these tumor cells left little time for the antibody to elicit its effect (Figure S4E, F). Taken together, these data provided evidence that senescent A549 cells expressing substantial LUNX expression on the cell membrane showed increasing antigenicity, and were sensitive to anti-LUNX antibody-mediated killing.

### Immunogenic senescence improves LUNX-targeting therapy in a model of lung cancer

**Fig. 6:**
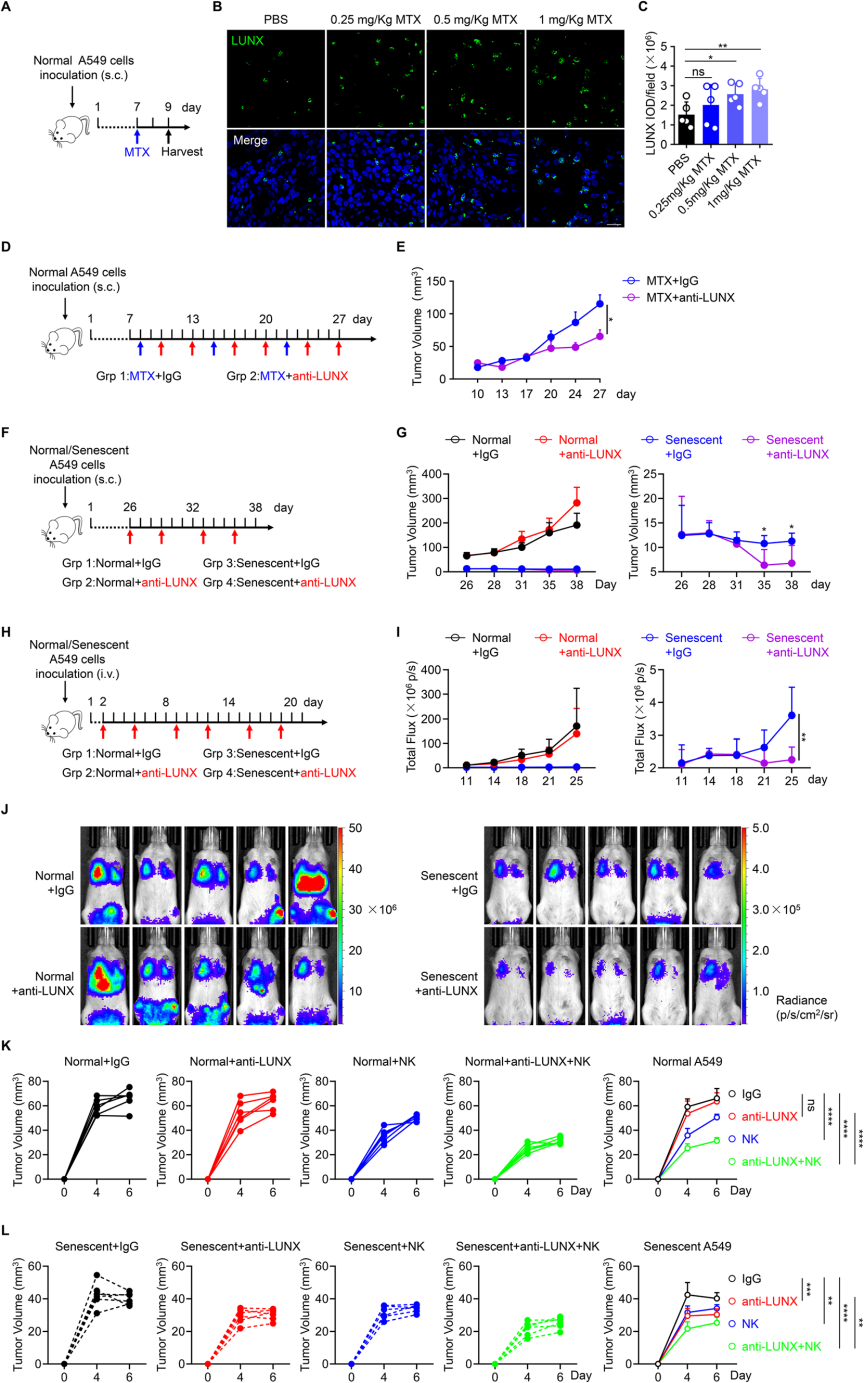
Immunogenic senescence improves LUNX-targeting therapy in a model of lung cancer (**A**–**C**) Mitoxantrone treatment improved LUNX expression *in vivo*. **A** Experimental scheme. A549 cells were injected (s.c.) and then mice were treated with the indicated concentration of mitoxantrone. Two days later, tumors were harvested for immunofluorescence detection of LUNX expression. Representative images are shown (**B**) and we calculated the intensity value of each field of LUNX (**C**). **D**, **E** Combination of mitoxantrone and anti-LUNX antibody (80 mg/kg) suppressed tumor growth. Schematic representation of the experimental protocol (**D**). Tumor-growth curves showed the mean tumor volume (mm^3^) ± SD (*n* = 5). **F**–**J** Immunogenic senescent A549 cells are sensitive to anti-LUNX antibody therapy. Schematic representation of the experimental protocol; the dose of anti-LUNX used is 80 mg/kg (**F**, **H**). Tumor-growth curves showing the mean tumor volume (mm^3^) ± SD (*n* = 6) (**G**) or the total flux (p/s) ± SD (*n* = 5) (I). Representative image of tumor burden in each mouse was shown in (**j**). **K**, **L** ADCC-mediated by NK cells suppressed tumor growth. 2 × 10^6^ normal (**K**) or senescent (**L**) A549 cells were inoculated subcutaneously with or without 1 × 10^6^ human PBMC derived NK cells in the presence of 500 μg/mL anti-LUNX antibody or human IgG. Tumor-growth curves in the right showing the mean tumor volume (mm^3^) ± SD (*n* = 6)

We wished to discover if the results mentioned above were translated *in vivo*. We conducted a study based on inhibition of tumor growth with a xenograft model using A549 cells. First, we injected (s.c.) normal A549 cells into immune-deficient nude mice. We treated these tumor-bearing mice with increasing doses of mitoxantrone. Two days later, we harvested the tumors for immunofluorescence-based measurement of LUNX expression (Fig. 6A–C). As expected, mitoxantrone treatment increased the cell surface expression of LUNX within the tumor bed *in vivo*. Then, we tested if a combination of mitoxantrone with an anti-LUNX antibody led to efficient tumor control. Co-treatment with mitoxantrone and an anti-LUNX antibody caused suppression of subcutaneous tumor growth as evidenced by a significant reduction in tumor volume when compared with the group treated with mitoxantrone alone (Fig. 6D, E). These results suggested that sequential combination of mitoxantrone and anti-LUNX antibody might increase the expression of LUNX and kill LUNX-expressing cells, respectively, and finally lead to reduced tumor progression.

Next, we wondered whether normal cells and senescent A549 cells showed the same sensitivity to an anti-LUNX antibody *in vivo*. NOD.SCID γc-deficient mice were inoculated (s.c.) with normal cells or senescent A549 cells via the subcutaneous (Fig. 6F, G) or intravenous (Fig. 6H, I) route. Notably, normal A549 cells proliferated much faster than senescent A549 cells *in vivo*, and the latter showed negligible growth irrespective of where the tumor cells were inoculated (Fig. 6G, I). This difference was unsurprising because senescent A549 cells barely proliferated *in vitro*. Furthermore, senescent A549 cells were much more sensitive to anti-LUNX antibody because mice bearing this type of tumor cell had a lower tumor burden compared with mice in the IgG group (Fig. 6G, I). Live images of mice also showed a much lower tumor burden in mice bearing senescent A549 cells and then treated with anti-LUNX antibody (Fig. 6J). To further prove the importance of NK cells-mediating-ADCC in tumor rejection, we subcutaneously inoculated normal or senescent A549 cells with or without human PBMC derived NK cells in the presence of anti-LUNX antibody or human IgG. For both normal and senescent A549 cells, inoculation with anti-LUNX antibody in the presence of NK cells significantly reduced the tumor volume compared to inoculation with anti-LUNX antibody alone, which suggested that ADCC-mediated by NK cells also contributed to tumor rejection *in vivo* (Fig. 6K, L). As shown in Fig. 6K, L, senescent A549 cells were much more sensitive to anti-LUNX antibody as they showed lower tumor volume when inoculated with anti-LUNX antibody compared to inoculation with NK cells, which were not seen in normal A549 cells.

Our aforementioned results (Figure S1C, 1D) suggested that gemcitabine could also induce the translocation of LUNX. Accordingly, we tested the anti-tumor ability of gemcitabine combined with anti-LUNX antibody *in vivo*. As expected, gemcitabine also showed an immunogenic senescence effect because anti-LUNX antibody-treated mice showed a lower tumor burden and slower tumor progression (Figure S5A–C). Taken together, these results indicated that immunogenic drugs that promoted LUNX shuttling might improve LUNX-targeting therapy and inhibit tumor growth.

## Discussion

We propose that induction of immunogenic senescence of tumor cells is a promising approach for cancer immunotherapy. Tumor cells develop multiple adaptations to the immune system to escape recognition, but their immunogenicity can be strengthened through the induction of senescence [28]. Senescence has been thought to serve as a tumor-suppressive mechanism [18, 26]. Kang and colleagues showed that senescence surveillance prevents the expansion of premalignant cells in hepatocellular carcinoma [24]. Likewise, senescent tumor cells strongly activate NK cells through NKG2D or other activating receptors and, finally, are eliminated by the immune system in multiple myeloma [7, 39]. Recently, Amor and colleagues identified that urokinase-type plasminogen activator receptor-specific CAR T cells can efficiently ablate senescent cells in senescence-associated diseases not limited to tumors (e.g., liver fibrosis) [1]. Thus, senescent tumor cells coincide with a more immunogenic phenotype than their normal-cell counterparts and are distinguished by much higher expression of common activating ligands and specific tumor antigens.

We demonstrated that LUNX is a lung cancer targetable tumor antigen during immunogenic senescence. Targeting LUNX via anti-LUNX antibody suppressed the survival of senescent tumor cells *in vitro* and *in vivo*. We showed that LUNX expression on the cell surface can be increased significantly by immunogenic chemotherapy using mitoxantrone, which induces a senescence phenotype instead of immunogenic cell death as reported previously [30, 40]. This difference in data may have resulted from the different doses used in our study. Tumor cells are not killed directly by a sublethal dose of mitoxantrone treatment, but most of these cells enter “premature senescence”, which is strengthened further even after drug withdrawal. Attempting to survive under this drug-induced stress, tumor cells express more LUNX on their plasma membrane, the overexpression of which we showed previously to promote the migration and proliferation of lung cancer cells [43]. Targeting surface LUNX via anti-LUNX antibody at this time more efficiently suppresses the survival of these LUNX-high-expressing senescent tumor cells. Meanwhile, these cells also exhibit a stronger potential to activate the immune system with the expression of NKG2D ligands as well as the adjuvant molecule calreticulin [31, 35]. Though these senescent tumor cells survive sublethal stress, they remain in a highly immunogenic state with strong antigenicity and adjuvanticity.

Reports have suggested that senescent cells show high expression of NKG2D ligands (e.g., MIC-A and ULBP2) and are sensitive to NKG2D ligand-dependent killing [21, 35]. This phenomenon may be dependent upon the intrinsic low expression of these activating ligands. We also detected increased expression of these ligands on senescent tumor cells. However, there was no significant difference in the sensitivity to NK cell-mediated killing because these tumor cells had a higher background expression than that of normal cells. Our recent report demonstrated that low-dose gemcitabine treatment could enhance the immunogenicity of lung cancer cells [41]. Gemcitabine treatment also increased the expression of LUNX on cell surfaces *in vitro* and suppressed tumor growth *in vivo*, so additional studies will be needed to accelerate the translation from basic science research to the clinic. Our findings suggest that the induction of immunogenic senescence using FDA-approved drugs in combination with anti-LUNX antibody might provide a new strategy for NSCLC treatment.

### Supplementary Information

Below is the link to the electronic supplementary material.

Supplementary file1 (PDF 24832 KB)

## Data Availability

All data are available.
