# Supplementary material for: Immunogenic senescence sensitizes lung cancer to LUNX-targeting therapy
Source: Cancer Immunol Immunother. 2021 Oct 21;71(6):1403–17. doi: 10.1007/s00262-021-03077-1 (PMC9123058; doi:10.1007/s00262-021-03077-1)
Supplement: Supplementary file 1 — Supplementary file1 (PDF 24832 KB) [file 262_2021_3077_MOESM1_ESM.pdf]

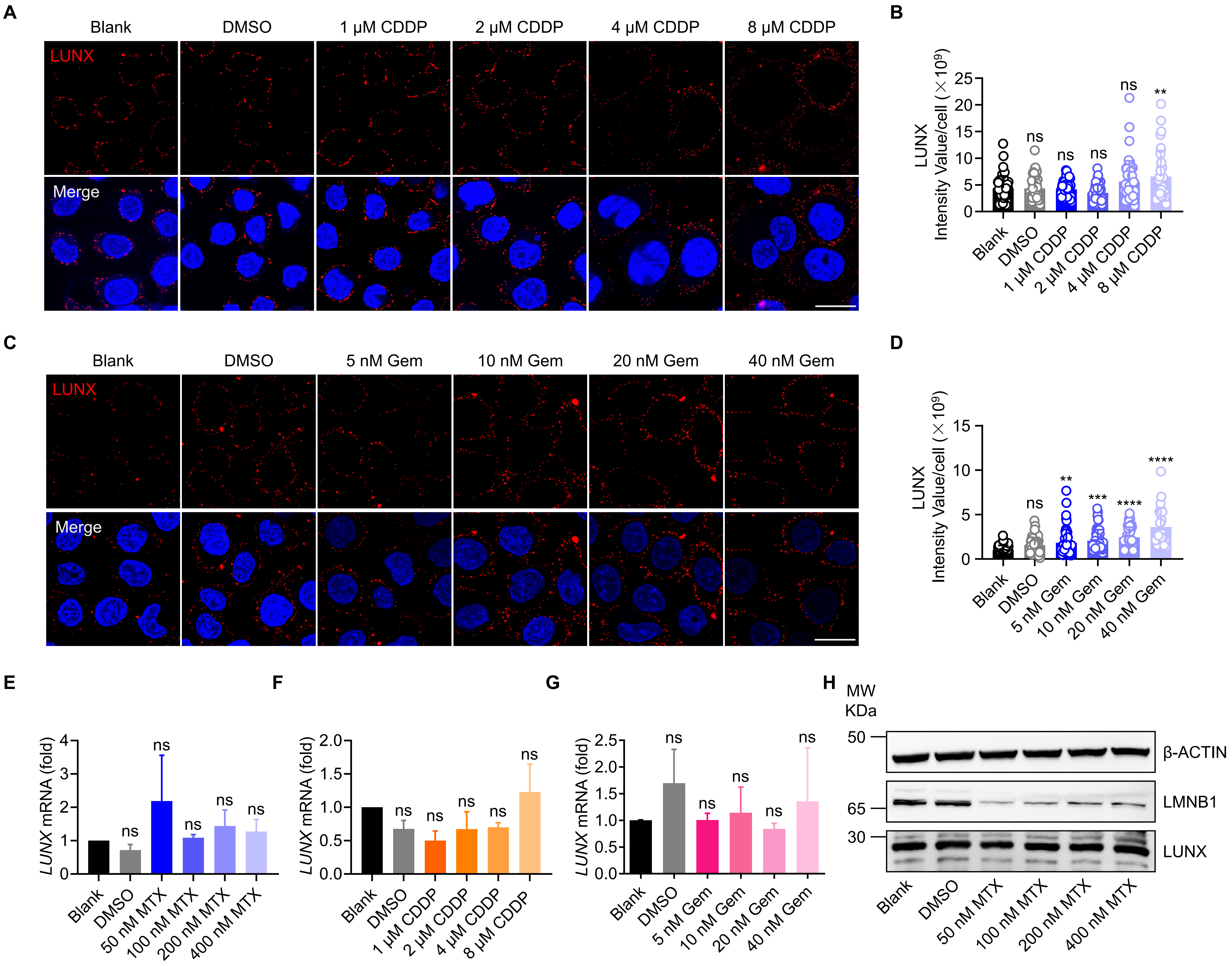

Figure S1. LUNX Expression after Chemotherapy Treatment (Related to Fig. 1)
(A-D) Immunofluorescence detecting surface LUNX expression of NCI-H292 cells. Representative images are shown for cisplatin (CDDP) treatment (A) and Gemcitabine (Gem) treatment (C). The scale bar = 20 μm. Anti-LUNX concentration = 200 μg/mL. Statistics calculated by the intensity value of each cell for CDDP treatment (B) and Gemcitabine treatment (D).
(E-G) Quantitative RT-PCR of LUNX mRNA in A549 cells treated with mitoxantrone (E), CDDP (F) and Gemcitabine (G).
(H) Lysates of whole A549 cells were collected 48 h after treatment with mitoxantrone and analyzed by western blotting using the indicated antibodies. Actin is shown as a loading control. Anti-LUNX concentration = 200 μg/mL.

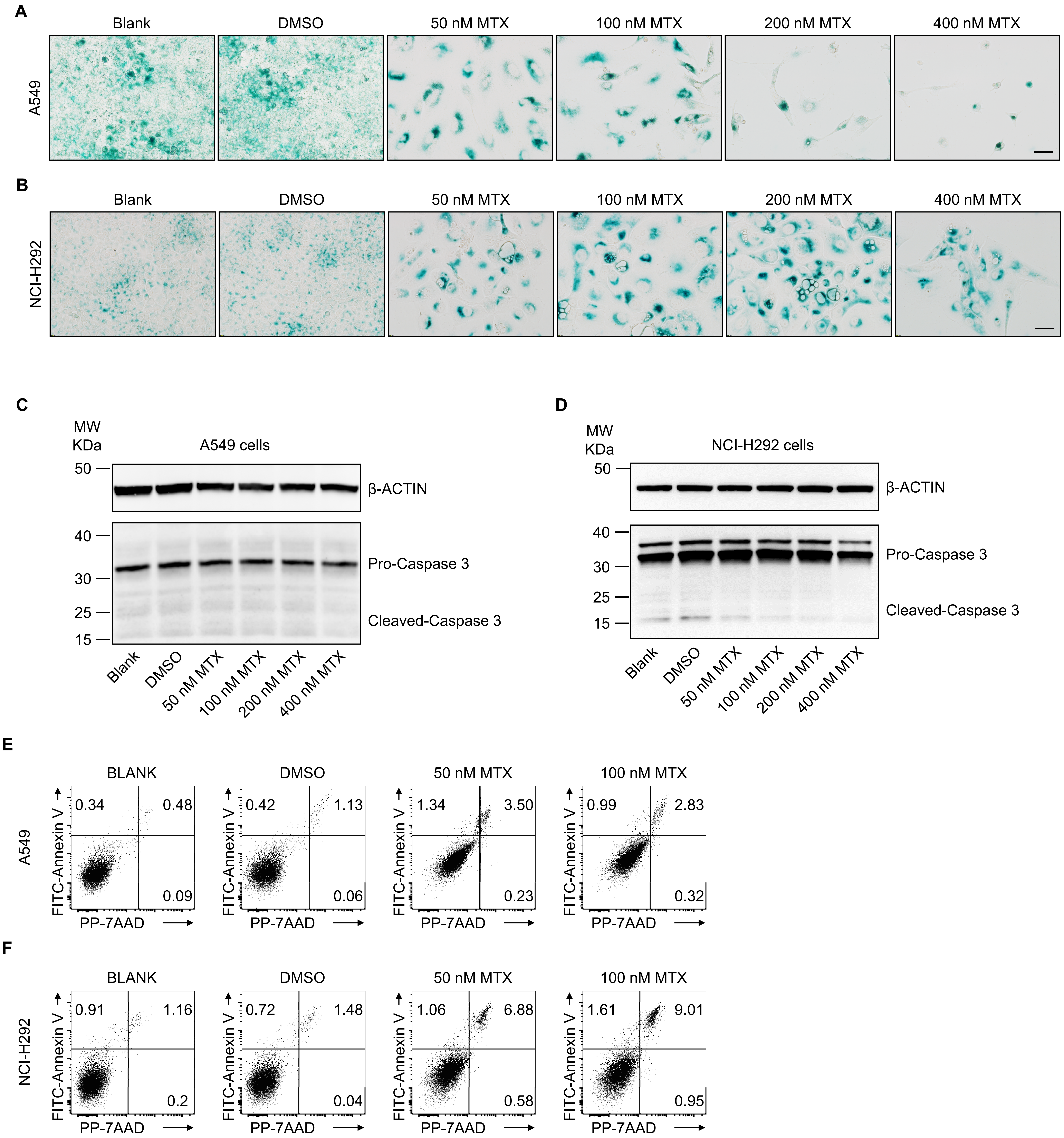

Figure S2. Mitoxantrone Treatment Induced A549 cells Senescence (Related to Fig. 2)

(A-B) A549 and NCI-H292 cells were assayed for SA-b-gal activity 7 days after withdraw the treatment with the appropriate concentration of mitoxantrone for 48 hours. Representative images are shown for A549 (A) and NCI-H292 (B). The scale bar = 50 μm.

(C-D) Lysates of whole A549 cells and NCI-H292 cells were collected 48 h after treatment with mitoxantrone and analyzed by western blotting using the caspase 3 antibodies. Actin is shown as a loading control.

(E-F) FACS analysis of annexin V and 7AAD expression of A549 cells (E) and NCI-H292 cells (F) after the treatment of indicated concentration of mitoxantrone for 48h.

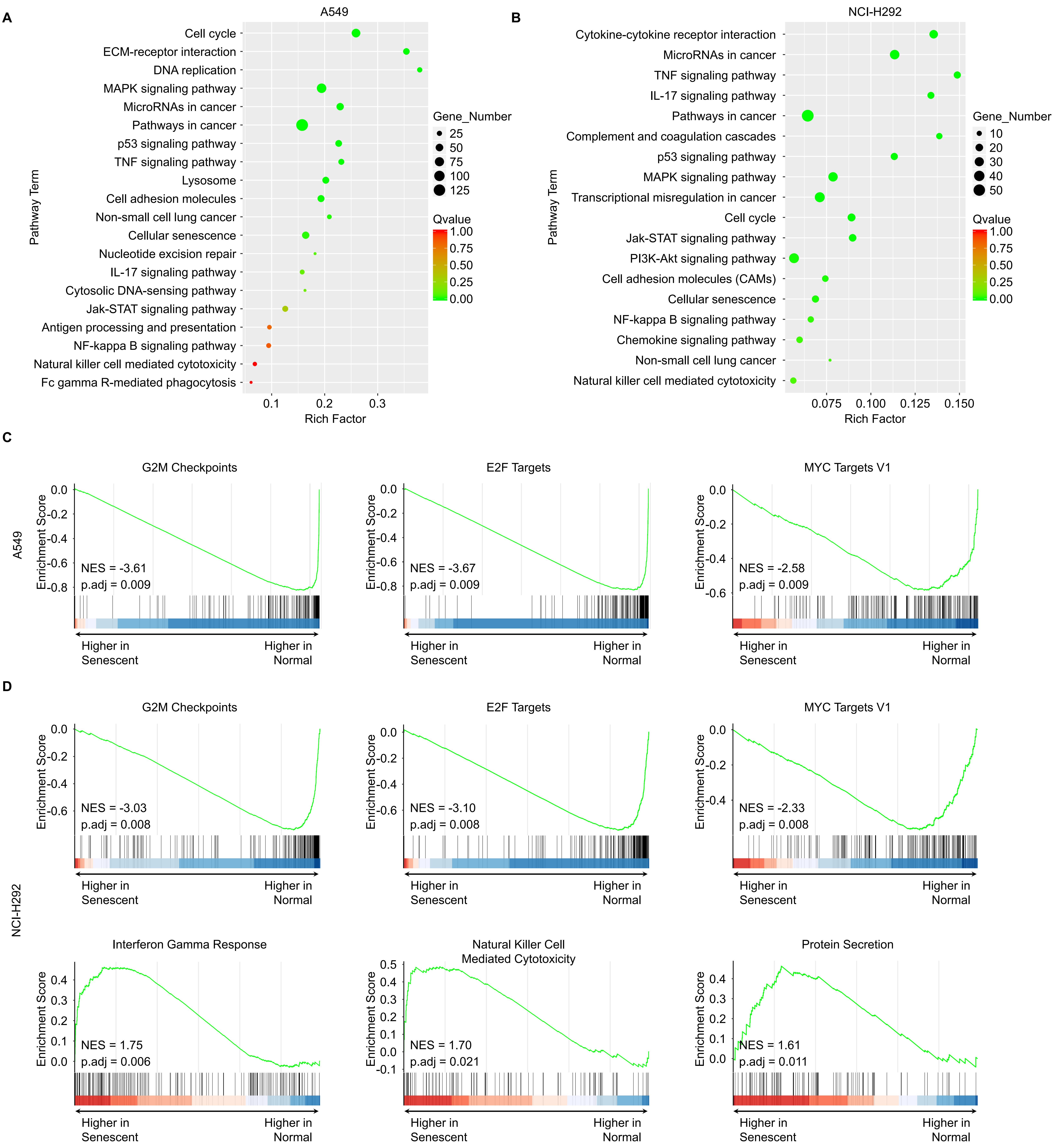

Figure S3. Senescent Cells Exhibit Immunogenic Signature (Related to Fig. 4)
(A-B) KEGG analysis of the differentially expressed genes was performed to evaluate enriched biological processes for A549 (A) and NCI-H292 cells (B).
(C-D) Significantly enriched pathways by GSEA analysis comparing senescent A549 cells versus normal A549 cells (C) and senescent NCI-H292 cells versus normal NCI-H292 cells (D).

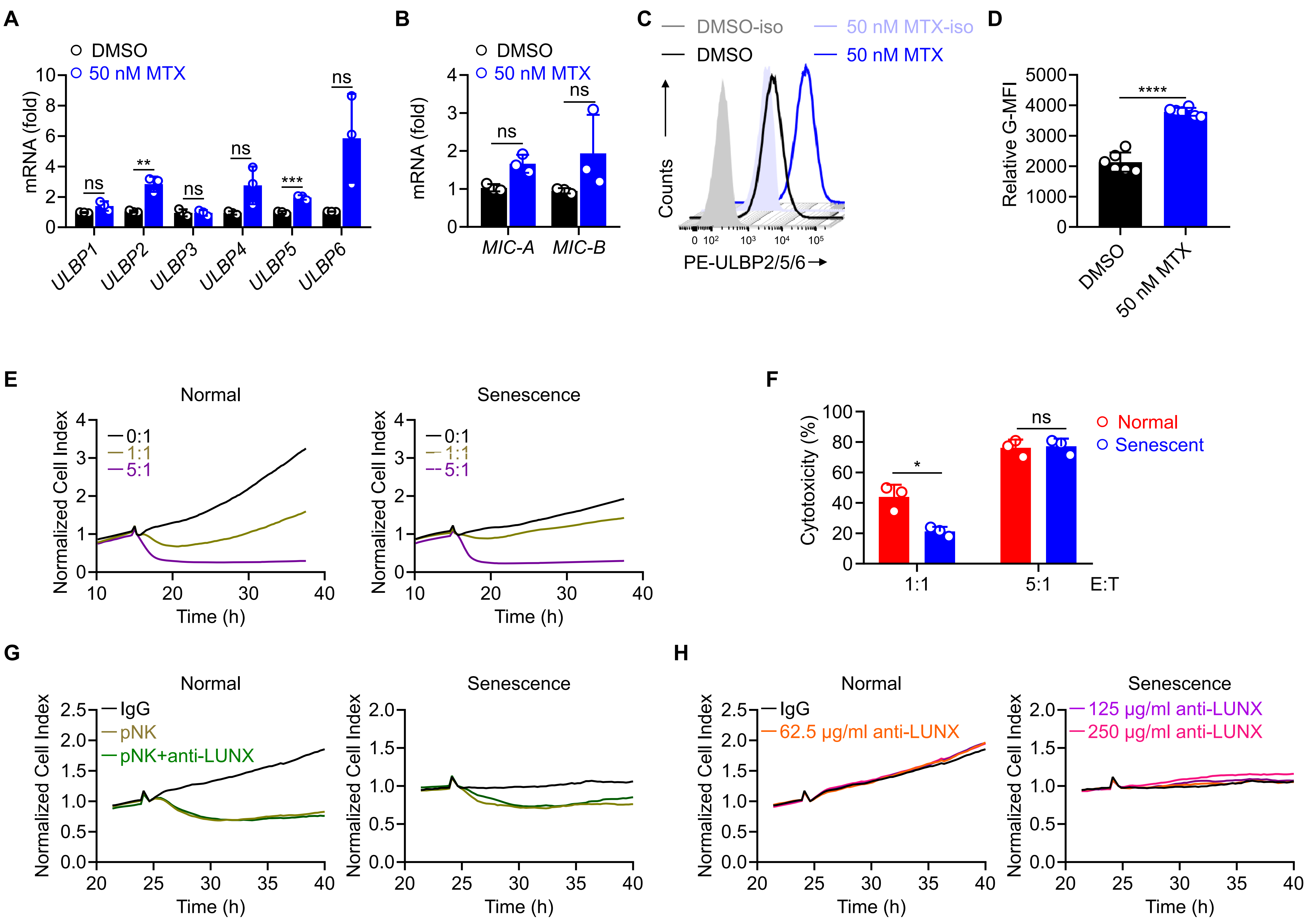

Figure S4. NCI-H292 Cells were Refractory to anti-LUNX Antibody (Related to Fig. 5)
(A-B) Quantitative RT-PCR of NKG2D ligands genes in NCI-H292 cells treated with mitoxantrone or DMSO.
(C-D) Flow cytometry of ULBP2/5/6 expression in NCI-H292 cells treated with mitoxantrone or DMSO. Statistics calculated by the relative geometric-MFI of NCI-H292 cells treated with mitoxantrone or DMSO.
(E-F) Real-time cell index measurement of normal or senescent NCI-H292 as target cells cultured with different ratios of effector NK cells. The relative cytotoxicity is shown in (F).
(G) Real-time cell index measurement of normal or senescent A549 target cells cultured with ratio 2:1 of effector NK cells in the presence or absence anti-LUNX antibody. Anti-LUNX concentration = 200 μg/mL.
(H) Real-time cell index measurement of normal or senescent A549 target cultured in the presence of different concentrations of anti-LUNX antibody.

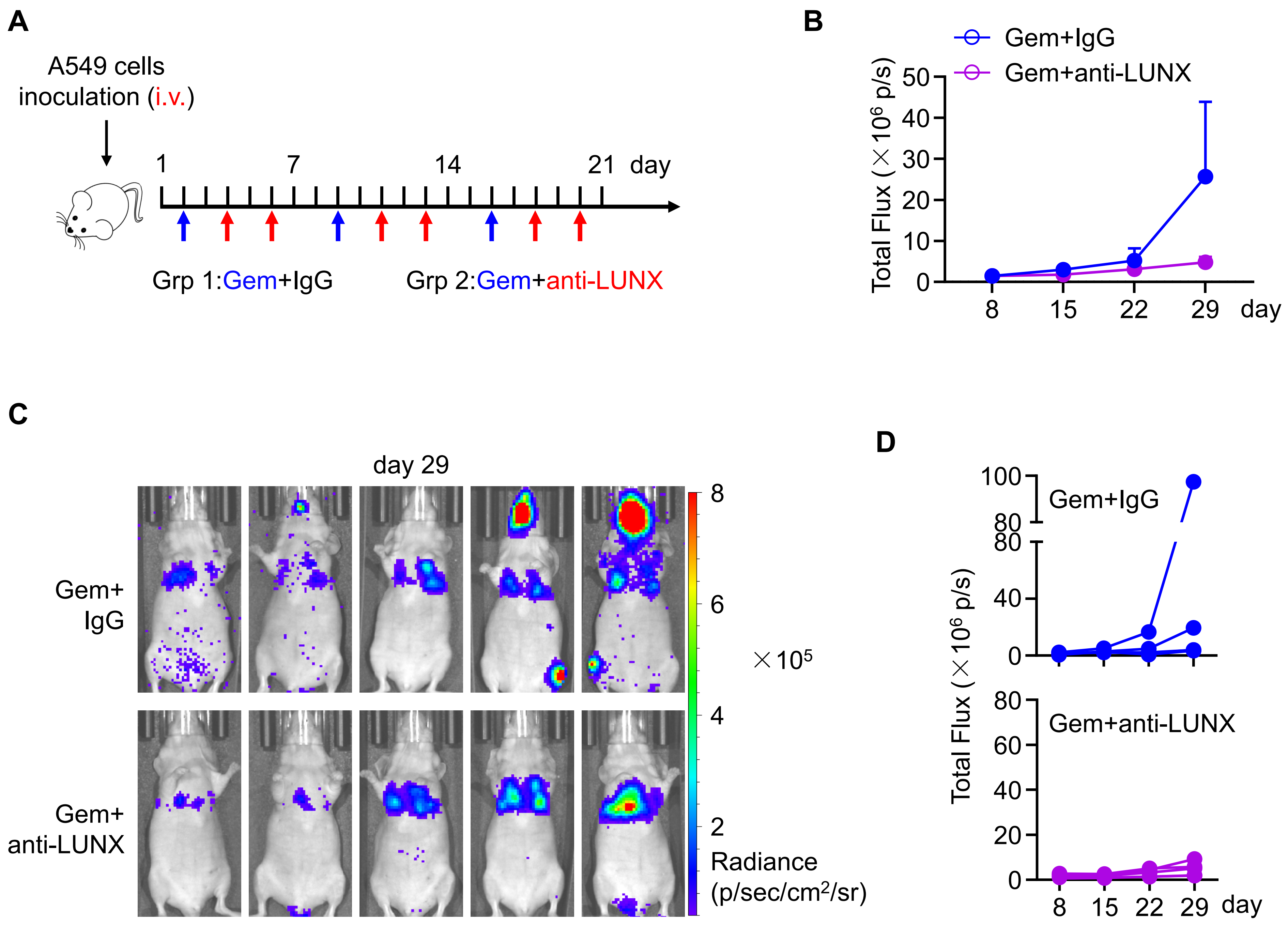

Figure S5. Gemcitabine Treatment Improves LunX-Targeting Therapy against Lung Cancer Models (Related to Fig. 6)
(A) Schematic representation of the experimental protocol.
(B-D) Tumor growth curves showing the total flux (p/s) ± SD (n = 5) (B). Representative image of tumor burden in each mouse (C) and Tumor growth curves of each mouse. Anti-LUNX concentration = 80 mg/kg.
